# Structural and functional hyperconnectivity within the sensorimotor system in xenomelia

**DOI:** 10.1002/brb3.657

**Published:** 2017-02-23

**Authors:** Jürgen Hänggi, Deborah A. Vitacco, Leonie M. Hilti, Roger Luechinger, Bernd Kraemer, Peter Brugger

**Affiliations:** ^1^ Division NeuropsychologyDepartment of PsychologyUniversity of ZurichZurichSwitzerland; ^2^ Neuropsychology UnitDepartment of NeurologyUniversity Hospital ZurichZurichSwitzerland; ^3^ Institute for Biomedical EngineeringUniversity and ETH ZurichZurichSwitzerland; ^4^ Psychiatric ServicesHospitals of the Canton of SolothurnOltenSwitzerland; ^5^ Center for Integrative Human Physiology (ZIHP)University of ZurichZurichSwitzerland

**Keywords:** body integrity identity disorder, diffusion tensor imaging, limb amputation, resting state functional magnetic resonance imaging, sensorymotor system, structural and functional hyperconnectivity

## Abstract

**Introduction:**

Xenomelia is a rare condition characterized by the persistent and compulsive desire for the amputation of one or more physically healthy limbs. We highlight the neurological underpinnings of xenomelia by assessing structural and functional connectivity by means of whole‐brain connectome and network analyses of regions previously implicated in empirical research in this condition.

**Methods:**

We compared structural and functional connectivity between 13 xenomelic men with matched controls using diffusion tensor imaging combined with fiber tractography and resting state functional magnetic resonance imaging. Altered connectivity in xenomelia within the sensorimotor system has been predicted.

**Results:**

We found subnetworks showing structural and functional hyperconnectivity in xenomelia compared with controls. These subnetworks were lateralized to the right hemisphere and mainly comprised by nodes belonging to the sensorimotor system. In the connectome analyses, the paracentral lobule, supplementary motor area, postcentral gyrus, basal ganglia, and the cerebellum were hyperconnected to each other, whereas in the xenomelia‐specific network analyses, hyperconnected nodes have been found in the superior parietal lobule, primary and secondary somatosensory cortex, premotor cortex, basal ganglia, thalamus, and insula.

**Conclusions:**

Our study provides empirical evidence of structural and functional hyperconnectivity within the sensorimotor system including those regions that are core for the reconstruction of a coherent body image. Aberrant connectivity is a common response to focal neurological damage. As exemplified here, it may affect different brain regions differentially. Due to the small sample size, our findings must be interpreted cautiously and future studies are needed to elucidate potential associations between hyperconnectivity and limb disownership reported in xenomelia.

## 1 Introduction

Xenomelia (McGeoch et al., 2011) is an unusual and rare condition characterized by the persistent and compulsive desire for the amputation of one or more physically healthy limbs. Individuals suffering from xenomelia typically report that their unwanted limb does not belong to their body and that they would feel “more complete” after its removal (Blanke, Morgenthaler, Brugger, & Overney, 2009). While it is hard to empathize with these feelings and inferences, extensive psychiatric examination of persons with xenomelia revealed a normal mental status and especially the absence of any psychotic disorder (First, 2005; Hilti et al., 2013; van Dijk et al., 2013). A recent review (Brugger, Lenggenhager, & Giummarra, 2013) covering telephone‐ and internet‐based interview studies in well over 100 persons with the disorder summarized the main findings: (1) The majority of affected persons are men (>90%); (2) there is an association of the disorder with nonheterosexuality, and a frequently accompanying symptom involves the erotic attraction by amputees; (3) adverse feelings linked to the unaccepted limb were usually noted in early childhood or adolescence; and (4) unaccepted limbs involve more frequently the lower limbs (>80%) and, in unilateral cases, there is a marked asymmetry in favor of left‐sided limbs (66%–80%; First, 2005; Hilti et al., 2013; Johnson, Liew, & Aziz‐Zadeh, 2011).

This latter observation, that more left‐sided than right‐sided limbs are the target of the amputation desire, has made some authors propose that xenomelia would primarily be a neurological disorder (McGeoch et al., 2011). In clinical neurology, transient feelings of nonbelonging of an own limb (as in hemiasomatognosia or somatoparaphrenia) or of hostility toward own body parts (as in misoplegia) are often‐described peculiarities of body‐self relations (Brugger, 2007; Critchely, 1953; Loetscher, Regard, & Brugger, 2006). As a matter of fact, these disorders are typically seen after right hemisphere lesions and accordingly involve the left side of the body with a much higher frequency (Brugger & Lenggenhager, 2014; Vallar & Ronchi, 2009). Stimulated by these reflections, Brang, McGeoch, & Ramachandran (2008) were the first who set out to seek empirical evidence for a neurological account of xenomelia. They applied pinprick to two individuals suffering from the disorder and found that stimulation of undesired compared to accepted parts of their body elicited an abnormally strong galvanic skin response. This was interpreted as the consequence of a pathologically exaggerated sympathetic outflow from limbic areas, notably the insula, to the parietal cortex. Such pathological responsivity might arguably have interfered, during early development, with the formation of a proper representation of the unwanted limb. The desire for amputation would thus be the consequence of an underrepresentation of a body part despite intact primary sensory and motor functions. In a subsequent study with four persons with xenomelia, the same author group recorded the magnetoencephalographic signal to tactile stimulation below and above the line of desired amputation (McGeoch et al., 2011). In contrast to touch on accepted body parts, touch on the undesired limb failed to elicit a cortical response in one particular brain area, that is, the right superior parietal lobule (SPL). This part of the parietal lobe is known to be core for establishing and maintaining a coherent sense of having a body (Berlucchi & Aglioti, 2010; Giummarra, Gibson, Georgiou‐Karistianis, & Bradshaw, 2008; Moseley, Gallace, & Spence, 2012). Intriguingly, a recent structural imaging study in participants with xenomelia (Hilti et al., 2013) identified neuroarchitectural abnormalities in exactly those cortical areas implicated by empirical research: the right anterior insular cortex (Brang et al., 2008) and the right SPL (McGeoch et al., 2011). Unexpectedly, these authors also found decreased cortical surface area in the right primary somatosensory cortex (SI) involved in the representation of the left leg, and in the right secondary somatosensory cortex (SII; Hilti et al., 2013).

To identify potential neuroarchitectural differences that could underlie the phenomenological feeling of “over‐completeness” reportedly characteristic of xenomelia (McGeoch et al., 2011), we set out to investigate structural and functional connectivity of the whole connectome. For that purpose, we compared 13 participants suffering from xenomelia with a carefully matched control group with respect to structural and functional connectivity using diffusion tensor imaging (DTI) combined with fiber tractography and resting state functional magnetic resonance imaging (rsfMRI), respectively. A whole‐brain connectome as well as region–of‐interest (ROI) approach have been applied to the DTI and rsfMRI data using advanced network‐based statistic tools. For the definition of the network nodes, we applied the commonly used automated anatomical labeling (AAL) ROI atlas (Tzourio‐Mazoyer et al., 2002) consisting of 116 different brain regions covering the whole cerebrum, cerebellum, and subcortical structures (whole‐brain connectome approach), but we also constructed a xenomelia‐specific network comprised by brain regions formerly shown to be anatomically altered in xenomelia compared with control men (Hänggi, Bellwald, & Brugger, 2016; Hilti et al., 2013) or differentially activated in different conditions in participants with xenomelia (van Dijk et al., 2013; ROI approach). We expected to find differences in structural and functional connectivity between subjects suffering from xenomelia and control men in brain regions belonging to the sensorimotor system and in the insula. We were also interested in finding further important brain regions associated with xenomelia.

## 2 Material and Methods

### 2.1 Subjects and study design

Thirteen men suffering from xenomelia were recruited via an Internet site. Aside from their desire for amputation, their medical history was free of any neurological and psychiatric disease, and thorough neurological and psychiatric examinations proved normal (Hilti et al., 2013; see Table 1 for details). A control group was separately recruited and did not differ from the xenomelia group with respect to handedness, footedness, age, and education. The individuals suffering from xenomelia, all desired an above knee amputation; eight of the left leg, two of the right leg and three of both legs. They completed the Zurich Xenomelia Scale (Aoyama, Krummenacher, Palla, Hilti, & Brugger, 2011), a 12‐item questionnaire designed to measure the degree of xenomelia and of associated symptoms like the erotic attraction by amputees and the urge to pretend to be an amputee. Scores were within the range reported for a group of 52 individuals with the disorder, who had filled in the questionnaire via Internet. For more details on participant characteristics, especially on the manifestations of xenomelia, see Table 1. All participants provided written informed consent. The study protocol was conducted in accordance with the Declaration of Helsinki and approved by the local ethics review board. DTI and resting state fMRI images were acquired in the same session.

**Table 1:** Demographic, xenomelia‐related, global brain, and psychiatric measures of the participants under investigation

	Participants with xenomelia (*n* = 13)		Control participants (*n* = 13)				
Demographic and xenomelia‐related measures	Frequency		Frequency				
Handedness (right/left/ambi)	12/1/0		12/1/0				
Footedness (right/left/ambi)	12/1/0		12/1/0				
Sexual orientation (hetero/homo/bi)	6/5/2		13/0/0				
Target leg (left/right/both)	8/2/3		n.a.				
Amputation desire since (years/I can remember)	7.86 y./2 p.		n.a.				
Height of desired amputation (AKA/BKA)	13/0		n.a.				
Triggering events (encounters with amputee(s)/no event)	9/4		n.a.				

	Mean	*SD*	Mean	*SD*	*t*‐value	*p*‐value	Effect size
Age, education, and global brain measures							
Age (years)	49.3	14.5	50.2	12.5	−0.16	0.87	−0.07
Education (years)	15.4	3.0	14.8	2.8	0.85	0.40	0.35
Total intracranial volume (cm^3^)	1,509.3	218.1	1,510.2	172.1	−0.01	0.99	−0.004
Total gray matter volume (cm^3^)	642.2	43.5	627.5	50.9	0.79	0.44	0.32
Total subcortical gray matter volume (cm^3^)	185.5	13.8	178.8	23.1	0.90	0.38	0.37
Total left white matter volume (cm^3^)	263.8	21.1	258.8	23.5	0.58	0.57	0.24
Total right white matter volume (cm^3^)	262.0	24.5	259.1	24.0	0.30	0.76	0.12
Total left cortical volume (cm^3^)	232.8	16.9	227.7	20.3	0.70	0.49	0.29
Total right cortical volume (cm^3^)	232.0	18.3	228.8	19.8	0.44	0.66	0.18
Total left cortical surface area (cm^2^)	856.9	52.9	849.3	60.5	0.34	0.74	0.14
Total right cortical surface area (cm^2^)	849.4	59.5	848.0	59.9	0.06	0.95	0.02
Mean left cortical thickness (mm)	2.462	0.110	2.452	0.078	0.27	0.79	0.11
Mean right cortical thickness (mm)	2.479	0.115	2.473	0.079	0.30	0.89	0.12
Xenomelia‐related and psychiatric measures							
ZXS—amputation desire	5.15	0.59	n.a.	n.a.	n.a.	n.a.	n.a.
ZXS—erotic attraction	4.73	1.12	n.a.	n.a.	n.a.	n.a.	n.a.
ZXS—pretending behavior	3.88	1.14	n.a.	n.a.	n.a.	n.a.	n.a.
ZXS—total score	4.59	0.61	n.a.	n.a.	n.a.	n.a.	n.a.
Body‐dysmorphic disorder	23.9	7.9	24.1	6.0	−0.77	0.94	−0.31
Borderline symptom list	63.8	27.9	44.6	22.5	1.93	0.07	0.79
Obsessive‐compulsive inventory, revised	11.9	5.9	11.0	10.2	0.28	0.78	0.11
Schizotypal personality questionnaire (SPQ‐G factor 1)	8.7	6.2	10.8	9.4	−0.69	0.50	−0.28
Schizotypal Personality Questionnaire (SPQ‐G factor 2)	13.5	9.2	16.0	11.2	−0.17	0.86	−0.07
Schizotypal personality questionnaire (SPQ‐G total)	4.8	5.1	5.2	3.8	0.61	0.55	0.25
Barratt impulsiveness scale	38.8	4.5	33.5	8.8	1.90	0.07	0.78
Depression anxiety stress scale	14.9	11.2	15.8	13.5	−0.19	0.85	−0.08
Dissociative symptoms	322.3	241.2	174.5	155.8	1.86	0.08	0.76
Personal attributes questionnaire (masculine/feminine)	43.3	2.7	42.1	3.4	1.03	0.31	0.42
Gender identity	59.2	8.2	60.2	8.9	−0.28	0.79	−0.11
Bem sex role inventory	219.2	17.1	216.8	21.5	0.32	0.75	0.13

Effect size has been computed according Cohen. AKA, above knee amputation; BKA, below knee amputation; ZXS, Zurich Xenomelia Scale.

### 2.2 Magnetic resonance imaging data acquisition

Scans were acquired on a 3.0 Tesla Philips Achieva whole‐body scanner (Philips Medical Systems, Best, The Netherlands) equipped with a transmit‐receive body coil and a commercial eight‐element sensitivity encoding (SENSE) head coil array. Two T1‐weighted images, resting state functional MRI (rsfMRI) spin‐echo echo‐planar imaging (EPI) scans, and diffusion‐weighted EPI scans were obtained for each participant.

A volumetric three‐dimensional T1‐weighted fast field echo sequence was applied twice to obtain two scans each with a duration of 468 s and a spatial resolution of 0.94 × 0.94 × 1.0 mm^3^ (acquisition matrix: 256 × 256 pixels, 160 slices). Further imaging parameters were field of view, FOV = 240 × 240 mm^2^; echo time, TE = 3.7 ms; repetition time, TR = 8.06 ms; flip angle = 8°, and sensitivity encoding (SENSE) factor = 2.1. The two scans were then coregistered and averaged to increase the contrast‐to‐noise ratio.

Resting state functional magnetic resonance imaging was applied while subjects rested quietly with closed eyes in the scanner. They were instructed to think of nothing in particular and to let their mind wander. Images of rsfMRI were acquired in the transversal plane with a spatial resolution of 2.5 × 2.5 × 4.0 mm^3^ (reconstructed 1.72 × 1.72 × 4.0 mm^3^). Imaging parameters were: TR = 4 s; TE = 35 ms; FOV = 220 × 220 mm^2^; slice thickness = 4 mm; number of slices = 40; SENSE factor = 1.8. The rsfMRI sequence lasted about 10 min (corresponding to 150 brain volumes).

Subsequently, two identical diffusion‐weighted sequences were applied with a spatial resolution of 2 × 2 × 2 mm^3^ (matrix: 112 × 112 pixels, 75 slices in transversal plane). Diffusion was measured along 32 noncollinear directions (*b* = 1,000 s/mm^2^) preceded by a nondiffusion‐weighted volume (reference volume, *b* = 0 s/mm^2^). Further imaging parameters were: Field of view, 224 × 224 mm^2^; echo time, 55.0 ms; repetition time, 13.010 s; flip angle, 90°; SENSE factor, 2.1. Scan time was about 8 min 42 s per sequence.

### 2.3 Data preprocessing for structural connectome analyses

Preprocessing of the diffusion‐weighted MRI data was performed with the FMRIB software library tools (FSL, version 5.0.6; http://www.fmrib.ox.ac.uk/fsl/; Smith et al., 2004) such as the FDT (FMRIB diffusion toolbox; version 3.0; Behrens et al., 2003). For deterministic fiber tractography, we used the Diffusion Toolkit (DTK, version 0.6.2.1) and TrackVis software (version 0.5.2.1; http://trackvis.org/; Park et al., 2009). The connectivity matrix was computed in MATLAB (version 8.0.0.783; http://www.mathworks.com/index.html).

To construct the connectivity matrix of the white matter pathways, the following fully automated preprocessing steps were realized: (1) In a first step, a binary brain mask was created using FSL's brain extraction tool. This mask was used in later steps to exclude non‐brain tissue. (2) Eddy current distortions and head movements were corrected using the EDDY_CORRECT tool of FDT. (3) Diffusion gradients were adjusted for rotations introduced by the eddy current and head movement corrections. (4) The preprocessed DTI data were then subjected to the DTK to compute voxel‐wise diffusion tensors and to construct the (principal) eigenvector and eigenvalue maps as well as a map of fractional anisotropy (FA). (5) Deterministic tractography was conducted in TrackVis using the “brute force” approach with an interpolated streamline‐tracking algorithm. Twenty streamlines per voxel were propagated (using the ‐rseed option in DTK with value 20) and fiber tracking was stopped if FA was lower than 0.10 or if the turning angle of a streamline between two consecutive voxels was larger than 45°. This resulted in a whole‐brain connectome comprised by about 2–3 million streamlines including subcortical pathways and connections to the cerebellum. (6) The individual FA map was registered onto the FMRIB58‐FA template, which is in correspondence with the MNI152 standard space, using FSL's linear image registration tool (FLIRT) and the resulting transformations were stored. (7) These transformations were then applied to the streamlines produced in step 6 to transform the streamlines into the MNI152 space. (8) The AAL ROIs (Tzourio‐Mazoyer et al., 2002), which are already in MNI152 standard space, were used to count the number of streamlines between each pair of ROIs. This AAL template consists of 116 ROIs covering the entire neocortex (78 cortical ROIs) and the subcortical structures amygdala, hippocampus, thalamus, caudate, putamen, and pallidum (12 subcortical ROIs) as well as 26 cerebellar ROIs. (9) Streamlines running through the brainstem and streamlines shorter than 5 mm in length were removed (denoted streamlines omitted in Table 1). Streamlines that make connections within a ROI itself were deleted (denoted selfloops). The number of the remaining streamlines between any pair of ROIs (denoted streamlines used to populate matrix) was counted using MATLAB scripts (Zalesky, Fornito, & Bullmore, 2010). (10) This procedure resulted in an undirected, weighted, 116 × 116‐connectivity matrix for each individual participant. The strength of a structural connection was operationalized by the number of reconstructed streamlines between two ROIs. (11) The undirected, weighted 116 × 116‐node connectivity matrices were then subjected to a network‐based statistical analysis (see below). We also investigated a smaller and more xenomelia‐specific network with 28 nodes (see below), the brain regions of which were derived from previous empirical neuroimaging findings in xenomelia (Hänggi et al., 2016; Hilti et al., 2013; van Dijk et al., 2013).

### 2.4 Data preprocessing for functional connectome analyses

Functional MRI data were preprocessed with DPARSFA toolbox (version 3.1) that is part of DPABI (version 1.2, http://rfmri.org/dpabi; Chao‐Gan & Yu‐Feng, 2010) using functions of SPM 8 (www.fil.ion.ucl.ac.uk/spm/software/spm8), comprising the following steps: (1) Coregistration of the T1‐weighted image onto the functional images, (2) slice timing correction, (3) realignment, (4) estimation of linear and nonlinear normalization of the T1‐weighted MRI image using the unified segmentation approach as implemented in SPM8, (5) estimated transformations were then applied to the functional images, (6) voxel resampling to 2 × 2 × 2 mm^3^, (7) smoothing with a Gaussian kernel of 6 mm full width at half maximum, (8) detrending, (9) regressing out the variance related to translations and rotations (using the Friston24 procedure) and the variance related to the white matter and cerebrospinal fluid signal. No mean global signal regression has been performed due to an ongoing dispute related to negative correlations observed after mean global signal regression (Qing, Dong, Li, Zang, & Liu, 2015; Wong, Olafsson, Tal, & Liu, 2012; Yeh, Tseng, Lin, Tsai, & Huang, 2015). (10) Data were filtered in the range between 0.01 and 0.1 Hz. (11) The strength of a functional connection was operationalized by the Fisher's *z*‐transformed Pearson correlation coefficient computed across the preprocessed time‐series between two ROIs. This procedure resulted in an undirected, weighted, 116 × 116‐node connectivity matrix for each individual participant that was then subjected to a network‐based statistical analysis (see below).

### 2.5 Regions‐of‐interest definition for the structural and functional xenomelia‐specific network analyses

A xenomelia‐specific 28‐node network has been constructed in addition. Sixteen nodes were derived from the eight clusters showing reduced cortical thickness or surface area in xenomelia compared with control men (Hilti et al., 2013). The 28‐node xenomelia‐specific network investigated in this study encompasses these 16 nodes in addition to the thalamus, caudate nucleus, putamen, and pallidum (Hänggi et al., 2016) as well as the ventral and dorsal premotor cortex (Blom et al., 2016; van Dijk et al., 2013). The eight subcortical nodes (bilateral thalamus, caudate nucleus, putamen, and pallidum) based on probability maps were derived from the Harvard–Oxford subcortical structural atlas (http://fsl.fmrib.ox.ac.uk/fsl/fslwiki/Atlases), whereas the remaining 20 nodes of the xenomelia‐specific 28‐node networks were spherical in nature and constructed using the MNI coordinates with a radius of 3 mm and 6 mm for the functional and structural analysis, respectively. These xenomelia‐specific 28‐node networks were used for both structural (with 6 mm radius spheres) and functional (with 3 mm radius spheres) network analyses. A larger radius for the spheres in the DTI analysis has been used to cover the white matter sufficiently. It is important to note that the findings reported by Hilti et al. (2013) and those reported by Hänggi et al. (2016) are based on the same experimental subjects as investigated in this study. Therefore, the construction of the xenomelia‐specific 28‐node network is not fully independent from the data previously reported (Hänggi et al., 2016; Hilti et al., 2013).

However, in addition to the independent AAL atlas (116‐node network analyses), some of the ROIs (i.e., ventral and dorsal premotor cortex) of the 28‐node xenomelia‐specific network were derived from the findings reported by van Dijk et al. (2013) and the subcortical structures thalamus, caudate nucleus, putamen and pallidum were derived from another independent brain atlas.

The functional network analyses have been performed on a whole‐brain basis as well as restricted to the edges that showed statistically significant differences in structural connectivity strength (DTI analysis) between the two groups. For that purpose, the strength of the functional connections between all pairs of nodes that were not involved in the structural subnetworks showing hyperconnectivity in xenomelia were set to zero in both the functional 116‐node and functional 28‐node connectivity matrices.

### 2.6 Network‐based statistical analyses

The network‐based statistical analyses of the structural (DTI) and functional (rsfMRI) connectivity data were performed using the network‐based statistic (NBS) tool (Zalesky et al., 2010; https://www.nitrc.org/projects/nbs/) using MATLAB (version R2013b, http://www.mathworks.com/). NBS is a method to control the family‐wise error rate when mass‐univariate testing is performed at every connection comprising the graph (network). NBS exploits the extent to which the connections comprising the contrast or effect of interest are interconnected. It is based on the principles underpinning traditional cluster‐based thresholding of statistical parametric maps (Zalesky et al., 2010).

On the basis of the general linear model approach, we used the *t*‐test module in NBS to compare the number of reconstructed streamlines of each connection (structural connectome) or the Fisher's *z*‐transformed correlation coefficient between two brain regions (functional connectome) of the 116‐ and 28‐node networks between the two groups. Comparisons were done with the *component extent* option in NBS that is suited for detecting an experimental effect that is relatively weak, but extends to encompass many connections. The numbers of streamlines of the significant connections (structural connectome) and the mean correlation (functional connectome) were extracted and averaged up over all significantly altered connections to correlate these values with each other.

For the NBS analyses based on the 116‐node network, we reported two network solutions using two different sensitivity thresholds (here, *t*‐thresholds), that is, a solution at a lower threshold (solution with many edges) and a solution at a higher threshold (solution with only few edges) for both contrasts (xenomelia < controls and xenomelia > controls). These *t*‐thresholds are not the actual alpha error probabilities. Rather they represent sensitivity (set) thresholds (Zalesky et al., 2010) and are used to determine which edges of the connectivity matrix form the largest subnetwork (suprathreshold component) that is subjected to the permutation statistic. These sensitivity thresholds must be determined by exploration and are therefore indeed chosen in an arbitrary way. However, this does not affect the false‐positive rate of the actual permutation statistic of the alpha error probability. Reporting findings based on selective *t*‐thresholds does not represent some sort of “cherry picking” because statistically significant subnetworks emerged at almost all set (sensitivity) thresholds explored. Therefore, we simply chose a lower threshold that provided a subnetwork of a size that is still interpretable, that is, not possessing too many connections at low thresholds. At high thresholds, we reported the threshold at which a statistically significant single subnetwork emerged, that is, we did not report subnetworks that disintegrated into different components at higher thresholds. It is important to note that in all analyses reported, we controlled the alpha error probability (*p* < .05) for multiple comparisons using 5,000 permutations of the group label. The permutation testing method implemented in NBS (Zalesky et al., 2010) is not new and is synonymous with conventional cluster‐based thresholding procedures of statistical parametric maps (Bullmore et al., 1999; Hayasaka & Nichols, 2004; Nichols & Holmes, 2002). *p*‐Values for the network‐based measures are reported one‐tailed due to the directed contrasts. Statistically significant subnetworks have been visualized using BrainNet Viewer software (https://www.nitrc.org/projects/bnv/; Xia, Wang, & He, 2013).

### 2.7 Other statistical analyses

Demographic, xenomelia‐related, psychiatric as well as global brain measures have been compared using *t*‐tests for independent samples. Pearson's correlation was applied to associate structural and functional connectivity measures to each other. These statistical tests were performed using IBM SPSS Statistics (version 22.0, http://www-01.ibm.com/software/analytics/spss/). If not otherwise stated, two‐tailed *p*‐values are reported.

## 3 Results

### 3.1 Demographic, xenomelia‐related, psychiatric, and global brain measures

Demographic, xenomelia‐related, psychiatric as well as global brain measures are summarized in Table 1. There were no significant differences in any of these variables. Three psychiatric questionnaires (borderline symptom list, Barratt impulsivity scale, dissociative symptoms) showed statistical trends (.05 < *p* < .10, uncorrected for multiple comparisons) toward higher values in participants with xenomelia compared with control men. However, items specifically asking for the rater's dissatisfaction with the own body or parts of it inflated the results of those questionnaires. These statistical tendencies would disappear if the xenomelia‐related items were removed so that no longer any trend toward an association between xenomelia and personality disorder can be observed, quite in correspondence to earlier findings in the literature (First, 2005; First & Fisher, 2012).

### 3.2 Structural 116‐node whole‐brain connectome analyses

**Figure 1:**
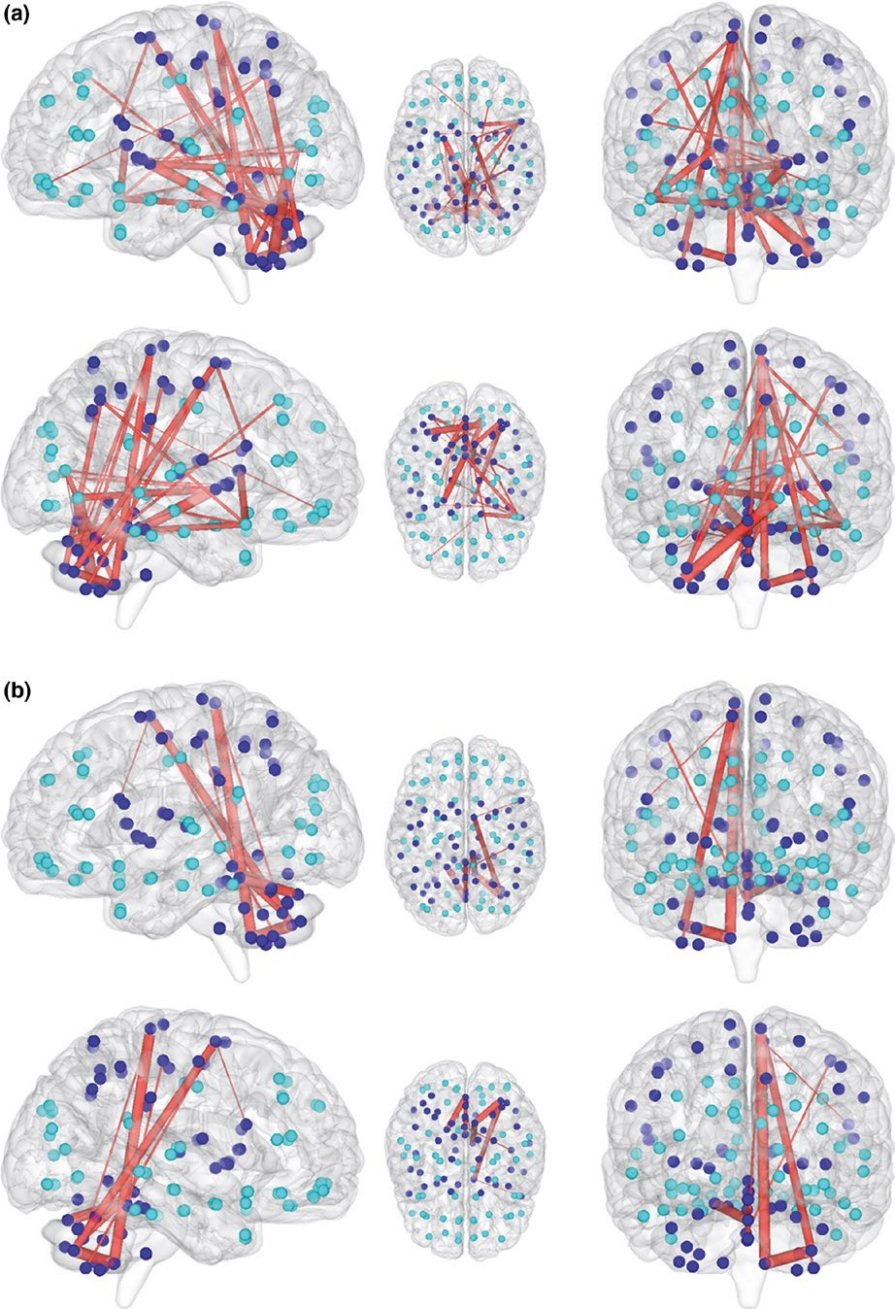
Increased structural connectivity in xenomelia. Shown are the results of the 116‐node network analysis. Two different solutions (sensitivity thresholds) are represented, one solution with many connections (a: *p* = .019, corrected for multiple comparisons) and one with less connections (b: *p* = .039, corrected for multiple comparisons). Blue circles represent nodes of the sensorimotor system, whereas turquoise circles represent all other nodes. The nodes are presented in Montreal neurological institute (MNI) space using the centroids of the regions of interest of the automated anatomical labeling (AAL) atlas. Red lines represent the white matter connections showing enhanced structural connectivity in xenomelia compared with control men

The network‐based statistical analyses of the 116‐node network revealed a subnetwork with statistically significantly increased structural connectivity in participants with xenomelia compared with control men (Figure 1, Table 2). With the lower sensitivity (set) threshold (*t* = 2.30), a subnetwork comprised by 35 connections distributed over 33 nodes has been revealed (*p* = .019, corrected for multiple comparisons, Figure 1a, Table 2). Most of these connections were realized among nodes belonging to the sensorimotor system such as the paracentral lobule (PCL), postcentral gyrus (PoCG), supplementary motor area (SMA), and parts of the cerebellum and these findings were, except for the cerebellum, clearly lateralized to the right hemisphere (Figure 1a, b, Table 2).

**Table 2:** White matter connections showing enhanced structural connectivity in participants with xenomelia compared with control subjects derived from the 116‐node network analysis

Node	Node	*t*‐value	Node	Node	*t*‐value
Both nodes within sensorimotor system			No or only one node within sensorimotor system		
Pallidum_L	Vermis_1_2	3.21	Lingual_R	Cerebellum_7b_L	3.30
**Cerebellum_Crus2_R**	**Cerebellum_9_R**	**2.97**	Caudate_R	Temporal_Pole_Sup_R	2.89
**Paracentral_Lobule_R**	**Cerebellum_9_R**	**2.92**	Lingual_R	Pallidum_L	2.88
**Cerebellum_4_5_L**	**Vermis_7**	**2.89**	Temporal_Pole_Sup_R	Vermis_1_2	2.81
**Supp_Motor_Area_R**	**Cerebellum_Crus2_R**	**2.83**	Precuneus_R	Cerebellum_Crus2_L	2.73
**Vermis_3**	**Vermis_8**	**2.70**	Calcarine_R	Cerebellum_9_L	2.71
**Vermis_3**	**Vermis_7**	**2.62**	Precuneus_R	Temporal_Pole_Sup_R	2.52
**Supp_Motor_Area_R**	**Cerebellum_7b_R**	**2.57**	Frontal_Sup_R	Vermis_8	2.48
**Postcentral_R**	**Vermis_10**	**2.56**	Hippocampus_R	Temporal_Pole_Sup_R	2.46
**Paracentral_Lobule_R**	**Vermis_8**	**2.55**	Calcarine_R	Pallidum_L	2.43
**Frontal_Inf_Oper_R**	**Supp_Motor_Area_R**	**2.52**	Calcarine_L	Pallidum_L	2.38
**Paracentral_Lobule_R**	**Vermis_10**	**2.49**	Frontal_Mid_Orb_L	Caudate_R	2.37
Paracentral_Lobule_R	Cerebellum_Crus2_L	2.44	ParaHippocampal_L	Vermis_7	2.37
Frontal_Inf_Oper_R	Thalamus_L	2.43	Calcarine_L	Putamen_L	2.34
Paracentral_Lobule_R	Cerebellum_Crus1_R	2.38	Precuneus_R	Caudate_L	2.33
Supp_Motor_Area_R	Vermis_8	2.36	Calcarine_R	Cerebellum_Crus1_L	2.31
Insula_R	Putamen_L	2.34	Hippocampus_R	Cerebellum_Crus1_L	2.30
			Calcarine_R	Vermis_1_2	2.30

Error probability was set at *p* < .05 corrected for multiple comparisons using 5,000 permutations of the group label (network‐based statistic tool). Bold printed connections are those found in both network solutions. Note that the connection from the left pallidum to the vermis 1,2 does not appear in the solution with the higher threshold because it becomes isolated from the subnetwork. Inf, inferior; L, left; Mid, middle; Oper, opercularis; Orb, orbitalis; R, right; Sup, superior; Supp, supplementary.

With the higher sensitivity threshold (*t* = 2.49), a subnetwork comprised by 11 connections distributed over 12 nodes has been found (*p* = .039, corrected for multiple comparisons; Figure 1b, Table 2). All nodes involved belong to the sensorimotor system and except for one node (cerebellum 4, 5 left), all nodes were within the right hemisphere.

Animations of the subnetworks presented in Figure 1a, b can be found online in the Supplementary Animations S1 and S2, respectively. No subnetworks with reduced structural connectivity in xenomelia compared with control men have been found.

### 3.3 Structural 28‐node xenomelia‐specific network analyses

**Figure 2:**
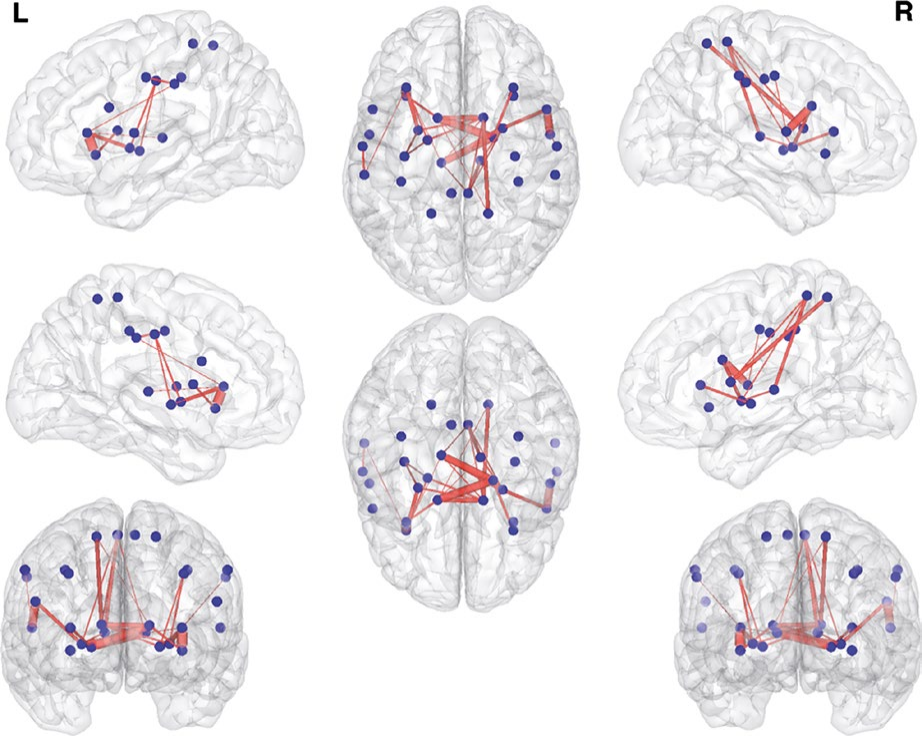
Increased structural connectivity in xenomelia. Shown are the results of the 28‐node xenomelia‐specific network analysis. Blue circles represent nodes of the sensorimotor system and the insula. These nodes were derived from three different xenomelia studies (Hänggi et al., 2016; Hilti et al., 2013; van Dijk et al., 2013). The nodes are presented in Montreal neurological institute (MNI) space using the centroids of the regions of interest derived from the above mentioned studies. Red lines represent the white matter connections showing enhanced structural connectivity in xenomelia compared with healthy control men. Note that this subnetwork was statistically significant only on a trend level (*p* = .087, corrected for multiple comparisons)

The network‐based statistical analyses of the 28‐node xenomelia‐specific network (Hilti et al., 2013; van Dijk et al., 2013) revealed a subnetwork with increased structural connectivity in participants with xenomelia compared with controls (Figure 2, Table 3). With a sensitivity threshold of *t* = 1.295, a subnetwork comprised by 28 connections distributed over 19 nodes has been found (Figure 2, Table 3), albeit only on a trend level toward statistical significance (*p* = .087, corrected for multiple comparisons).

**Table 3:** White matter connections showing enhanced structural connectivity in participants with xenomelia compared with control subjects derived from the 28‐node xenomelia‐specific network analysis

Node	Node	*t*‐value	Node	Node	*t*‐value
AIC_left_upper	AIC_left_lower	2.36	CS_left	Putamen_left	1.44
Caudate_left	Pallidum_right	2.19	Caudate_right	Putamen_left	1.44
S2_right	vPM_right	2.18	Putamen_right	Thalamus_right	1.42
Pallidum_right	Thalamus_left	2.07	AIC_left_upper	Pallidum_left	1.41
Putamen_right	Thalamus_left	1.80	S1_right	Caudate_right	1.40
Caudate_left	Caudate_right	1.74	CS_left	Pallidum_left	1.39
SPL_right	Caudate_right	1.68	S1_right	Putamen_right	1.38
AIC_left_upper	Putamen_left	1.65	Caudate_right	Thalamus_left	1.37
Putamen_right	vPM_right	1.62	S1_right	Thalamus_left	1.35
S1_right	Thalamus_right	1.61	S2_right	dPM_right	1.33
AIC_right_upper	Pallidum_right	1.53	IPL_left	AIC_left_upper	1.32
AIC_left_lower	Caudate_left	1.48	SPL_right	Caudate_left	1.31
IPL_left	dPM_left	1.46	AIC_left_upper	Thalamus_left	1.30
Caudate_right	Pallidum_left	1.45	Caudate_left	Thalamus_right	1.30

Error probability was set at *p* < .05 corrected for multiple comparisons using 5,000 permutations of the group label (network‐based statistic tool). Note that this subnetwork was statistically significant only on a trend level (*p* = .087, corrected). AIC, anterior insular cortex; CS, central sulcus; dPM, dorsal premotor cortex; IPL, inferior parietal lobule; L, left; Mid, middle; Oper, opercularis; Orb, orbitalis; R, right; S1 and S2, primary and secondary somatosensory cortex; SPL, superior parietal lobule; Sup, superior; Supp, supplementary; vPM, ventral premotor cortex.

An animation of the subnetwork presented in Figure 2 can be found online in the Supplementary Animation S3. No subnetworks with reduced structural connectivity in xenomelia compared with controls have been found.

### 3.4 Functional 116‐node whole‐brain connectome analyses

The functional network analyses have been performed using the full connectivity matrix information as well as using only the connections that showed statistically significant differences in structural connectivity strength between the two groups (see 3.2 above), setting all other connections in the connectivity matrix to zero.

**Figure 3:**
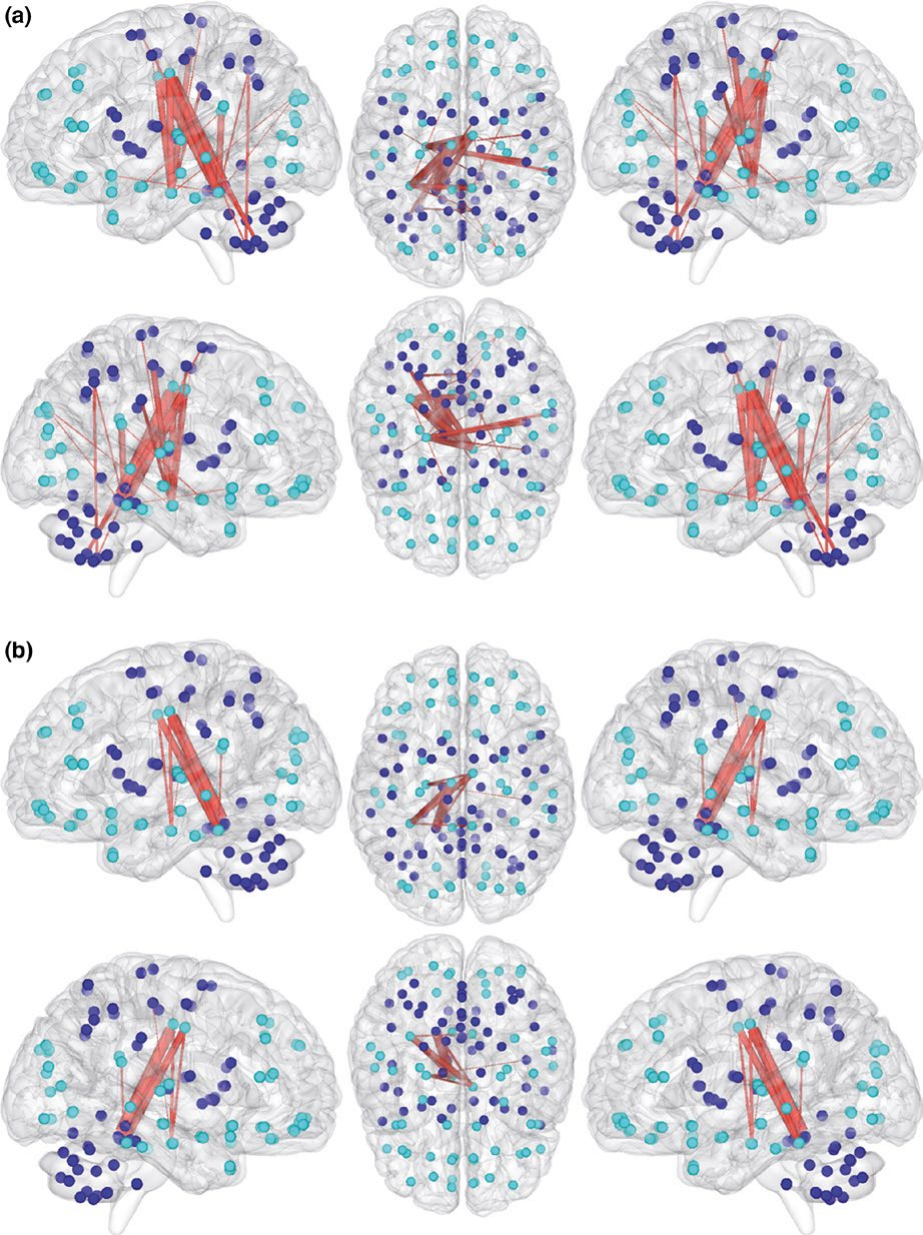
Increased functional connectivity in xenomelia. Shown are the results of the 116‐node network analysis. Two different solutions (sensitivity thresholds) are represented, one solution with many connections (a: *p* = .037, corrected for multiple comparisons) and one with less connections (b: *p* = .022, corrected for multiple comparisons). Blue circles represent nodes of the sensorimotor system, whereas turquoise circles represent all other nodes. The nodes are presented in Montreal neurological institute (MNI) space using the centroids of the regions of interest of the automated anatomical labeling (AAL) atlas. Red lines connect the nodes (brain regions) showing enhanced functional connectivity in xenomelia compared with healthy control men

The network‐based statistical analyses of the 116‐node network using the full connectivity matrix information revealed a subnetwork with increased functional connectivity in participants with xenomelia compared with control men (Figure 3, Table 4). With the lower sensitivity threshold (*t* = 3.60), a subnetwork comprised by 24 connections distributed over 20 nodes has been revealed (*p* = .037, corrected for multiple comparisons, Figure 3a, Table 4). Fourteen of these 24 connections were found among nodes belonging to the sensorimotor system with a preponderance of cerebellar nodes, but also including the right SMA, right PCL, right PoCG, and bilateral precentral gyrus (Figure 3a, Table 4). With the higher sensitivity threshold (*t* = 4.20), a subnetwork comprised by eight connections distributed over seven nodes has been found (*p* = .022, corrected for multiple comparisons, Figure 3b, Table 4). In this solution, the bilateral middle cingulate cortex encompassed most connections (Figure 3b, Table 4).

**Table 4:** Connections showing enhanced functional connectivity in participants with xenomelia compared with control subjects derived from the 116‐node network analysis

Node	Node	*t*‐value	Node	Node	*t*‐value
Both or one nodes within sensorimotor system			No node within sensorimotor system		
**Cingulum_Mid_L**	**Cerebellum_4_5_L**	**5.07**	**Cingulum_Mid_R**	**Fusiform_L**	**4.79**
**Cingulum_Mid_R**	**Cerebellum_4_5_L**	**4.48**	**Cingulum_Mid_L**	**Fusiform_L**	**4.61**
**ParaHippocampal_L**	**Postcentral_R**	**4.33**	**Cingulum_Mid_L**	**ParaHippocampal_L**	**4.51**
Cingulum_Mid_L	Cerebellum_7b_L	4.01	**Cingulum_Mid_R**	**ParaHippocampal_L**	**4.46**
ParaHippocampal_L	SupraMarginal_R	3.97	**Cingulum_Post_R**	**Fusiform_L**	**4.42**
Precuneus_R	Cerebellum_8_L	3.90	Fusiform_L	Precuneus_R	3.78
Cingulum_Mid_R	Cerebellum_7b_L	3.83	Cingulum_Post_R	Amygdala_R	3.70
Precentral_R	ParaHippocampal_L	3.81	Olfactory_L	Fusiform_L	3.68
Supp_Motor_Area_R	Cerebellum_7b_L	3.77	ParaHippocampal_L	Occipital_Sup_R	3.67
Cingulum_Mid_L	Cerebellum_8_L	3.75	Cingulum_Mid_R	ParaHippocampal_R	3.66
ParaHippocampal_L	Paracentral_Lobule_R	3.70			
Precentral_L	ParaHippocampal_L	3.64			
Occipital_Sup_R	Cerebellum_4_5_L	3.64			
Cingulum_Mid_R	Cerebellum_4_5_R	3.64			

Error probability was set at *p* < .05 corrected for multiple comparisons using 5,000 permutations of the group label (network‐based statistic tool). Bold printed brain regions (“functional connections”) are those found in both network solutions. L, left; Mid, middle; Post, posterior; R, right; Sup, superior; Supp, supplementary.

Animations of the subnetworks presented in Figure 3a, b can be found online in the Supplementary Animations S4 and S5, respectively. No subnetworks with statistically significantly reduced functional connectivity in participants with xenomelia compared with healthy control men have been revealed.

### 3.5 Functional 28‐node xenomelia‐specific network analyses

The network‐based statistical analyses of the 28‐node xenomelia‐specific network (Hänggi et al., 2016; Hilti et al., 2013; van Dijk et al., 2013) revealed no statistically significant subnetwork with neither enhanced nor reduced functional connectivity in participants suffering from xenomelia compared with healthy control men. However, if one accounts for the beta error probability by increasing the alpha error probability, there is border evidence (.13 < *p* < .24) for functional hyperconnectivity rather than hypoconnectivity also within this 28‐node xenomelia‐specific network (results not shown).

### 3.6 Functional connectivity within the structurally hyperconnected subnetworks

**Figure 4:**
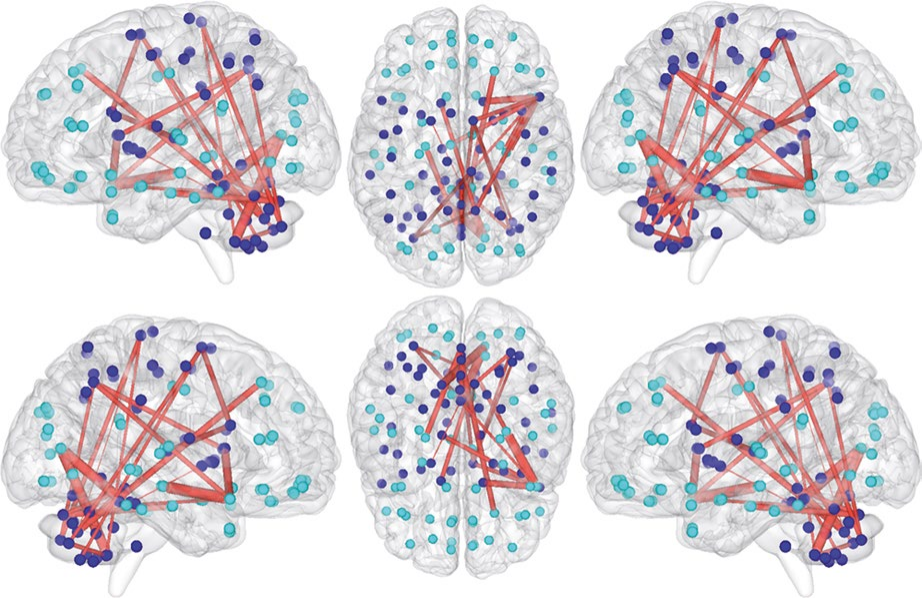
Increased functional connectivity within the structurally hyperconnected subnetwork. Shown are the results of the 116‐node functional network analysis restricted to the connections that already showed structural hyperconnectivity (see solution 1 in Figure 1 and Table 2). Blue circles represent nodes of the sensorimotor system, whereas turquoise circles represent all other nodes. The nodes are presented in Montreal neurological institute (MNI) space using the centroids of the regions of interest of the automated anatomical labeling (AAL) atlas. Red lines connect the nodes (brain regions) showing enhanced functional connectivity (*p* = .035, corrected for multiple comparisons) in xenomelia compared with healthy control men

The network‐based statistical analyses of the 116‐node network using only those connections of the connectivity matrix that were structurally altered between groups revealed a subnetwork with increased functional connectivity in participants with xenomelia compared with control men (Figure 4, Table 5).

**Table 5:** Enhanced functional connectivity in participants with xenomelia compared with control subjects derived from the 116‐node network analysis when restricting it to the structurally hyperconnected subnetwork (solution 1) shown in Figure 1 and Table 2

Node	Node	*t*‐value	Node	Node	*t*‐value
Both or one nodes within sensorimotor system			No node within sensorimotor system		
Calcarine_R	Vermis_1_2	1.93	Hippocampus_R	Temporal_Pole_Sup_R	2.09
Calcarine_R	Cerebellum_9_L	1.91	Precuneus_R	Temporal_Pole_Sup_R	1.13
Caudate_R	Temporal_Pole_Sup_R	1.58			
Frontal_Sup_R	Vermis_8	1.35			
ParaHippocampal_L	Vermis_7	1.32			
Vermis_3	Vermis_8	1.23			
Frontal_Inf_Oper_R	Thalamus_L	1.18			
Precuneus_R	Caudate_L	1.15			
Frontal_Inf_Oper_R	Supp_Motor_Area_R	1.13			
Paracentral_Lobule_R	Cerebellum_Crus2_L	1.06			
Cerebellum_Crus2_R	Cerebellum_9_R	1.02			
Supp_Motor_Area_R	Vermis_8	1.00			
Vermis_3	Vermis_7	0.99			
Precuneus_R	Cerebellum_Crus2_L	0.89			
Supp_Motor_Area_R	Cerebellum_Crus2_R	0.85			
Temporal_Pole_Sup_R	Vermis_1_2	0.81			
Supp_Motor_Area_R	Cerebellum_7b_R	0.80			
Paracentral_Lobule_R	Cerebellum_9_R	0.75			
Pallidum_L	Vermis_1_2	0.52			
Paracentral_Lobule_R	Vermis_8	0.42			
Cerebellum_4_5_L	Vermis_7	0.41			

Error probability was set at *p* < .05 corrected for multiple comparisons using 5,000 permutations of the group label (network‐based statistic tool). Inf, inferior; L, left; Mid, middle; Oper, opercularis; R, right; Sup, superior; Supp, supplementary.

With a sensitivity (set) threshold of *t* = 0.40, a subnetwork comprised by 23 connections distributed over 23 nodes has been revealed (*p* = .035, corrected for multiple comparisons, Figure 4, Table 5). Almost all of these connections were realized among nodes belonging to the sensorimotor system such as the PCL, SMA, thalamus, caudate nucleus, pallidum, and parts of the cerebellum and these findings were, with the exception of the subcortical structures, clearly lateralized to the right hemisphere (Figure 4, Table 5). No subnetworks with statistically significantly reduced functional connectivity in participants with xenomelia compared with healthy control men have been revealed.

Also, no statistically significant differences in functional connectivity within the 28‐node xenomelia‐specific network have been revealed between groups when focusing only on the connections of the 28‐node xenomelia‐specific subnetwork that already showed structural hyperconnectivity between groups (see 3.3 above).

### 3.7 Associations between structural and functional connectivity strength of the altered networks

For the structurally and functionally hyperconnected networks, we extracted the number of reconstructed streamlines and the mean correlation coefficient, respectively, for each connection and participant and averaged these values across all connections of the structural and functional networks separately. These connectivity strengths were then correlated with each other across both groups (Table 6).

**Table 6:** Associations between functional and structural connectivity strength of the altered networks

Mean connectivity strength in	116‐node rsfMRI network (solution 1)	116‐node rsfMRI network (solution 2)	116‐node rsfMRI network restricted to altered structural (DTI) connections
116‐node DTI network (solution 1)	*r* = .62, *p* = .0007	*r* = .66, *p* = .0002	*r* = .21, *p* = .30
116‐node DTI network (solution 2)	*r* = .47, *p* = .014	*r* = .52, *p* = .007	*r* = .17, *p* = .42
28‐node DTI network	*r* = .52, *p* = .006	*r* = .48, *p* = .012	*r* = .12, *p* = .57

Pearson correlations were computed across groups. DTI, diffusion tensor imaging; rsfMRI, resting state functional magnetic resonance imaging.

Mean structural connectivity strength of the solution 1 and 2 of the 116‐nodes structural network as well as those of the 28‐node structural network correlated positively with the mean functional connectivity strength of solution 1 and 2 of the 116‐nodes functional network (.47 < *r* < .66, .0002 < *p* < .014, respectively). Although the overlap of connections is the largest between the structural 116‐nodes network (solution 1) and the 116‐node functional network restricted to the connections showing structural hyperconnectivity, the structural and functional connectivity strengths of these two hyperconnected networks do not statistically significantly correlate with each other.

## 4 Discussion

This study reveals, to the best of our knowledge, first evidence for alterations in structural and functional brain connectivity in persons with xenomelia. Thirteen men suffering from an intense desire to have one leg (or both legs, *n* = 3) amputated were compared with a carefully matched control group. Building on previous reports of xenomelia‐related local structural and functional anomalies (Blom et al., 2016; Hänggi et al., 2016; Hilti et al., 2013; McGeoch et al., 2011; van Dijk et al., 2013), we here describe structurally and functionally hyperconnected sensorimotor networks including mainly the right‐hemispheric SPL, SI, SII, SMA, PCL, premotor cortex as well as the bilateral cerebellum.

After a brief paragraph on known functional and structural brain anomalies in persons with xenomelia, we first summarize the most important nodes of the hyperconnected networks found in this study in greater detail. Then we proceed to a discussion of the potential behavioral consequences of a structurally and functionally hyperconnected sensorimotor network considering the prominent clinical features of xenomelia. This discussion will also attempt to link the present findings to those of previous functional and structural neuroimaging studies, both in association with xenomelia and regarding other psychiatric conditions. After considering the limitations of this study, we will end with some educated speculations about the future status of xenomelia as a mental disorder with a circumscribed neurological pathophysiology.

### 4.1 Altered brain regions in xenomelia

This study was motivated by recently discovered alterations in circumscribed regions of the SPL, PCL, post‐ and precentral gyrus, dorsal and ventral premotor cortex, basal ganglia, thalamus, cerebellum, and insula as associated with xenomelia (Blom et al., 2016; Hänggi et al., 2016; Hilti et al., 2013; McGeoch et al., 2011; van Dijk et al., 2013). In addition to the ROI‐based approach that focused on the above‐mentioned sensorimotor brain regions, we also applied whole‐brain structural and functional connectome analyses to uncover potentially additional regions associated with the condition, but also to establish the specificity of the findings reported here.

One such region, the SPL, was found to be unresponsive to tactile stimulation of specifically those body segments that contribute to the feeling of “overcompleteness” and are therefore desired to be amputated (McGeoch et al., 2011). A consecutive morphometric study revealed reduced cortical thickness or surface area, not only in the right SPL, but also in right SI and right SII as well as in the right insula (Hilti et al., 2013). Evidence for the involvement of subcortical brain structures in xenomelia, hitherto not in the focus of neurologically motivated investigations, can be derived from a recently published study (Hänggi et al., 2016) conducted at our department and based on the same subjects as investigated in the present work. This study applied a vertex‐wise shape analysis that showed positive and negative tissue displacements (shape differences) in the form of thinning (hypotrophy) of bilateral dorsomedial putamina, left ventromedial caudate nucleus, and left medial pallidum in xenomelia compared with controls, whereas shape differences in the form of thickening (hypertrophy) of bilateral lateral pallida and left frontolateral thalamus in participants with xenomelia were also evident (Hänggi et al., 2016). This study provides strong evidence that in addition to the anatomical alterations already reported in the literature for cortical regions (Hilti et al., 2013; McGeoch et al., 2011), the subcortical structures thalamus, putamen, caudate nucleus, and pallidum might be related to xenomelia as well (Hänggi et al., 2016). A preliminary fMRI study based on five subjects suffering from xenomelia extended the above‐mentioned findings by suggesting that the ventral as well as dorsal premotor cortex are also brain regions importantly involved in generating the clinical picture of xenomelia, both from a functional (van Dijk et al., 2013) as well as structural point of view (Blom et al., 2016).

Although xenomelia has initially been conceptualized as a personality disorder and denoted body integrity identity disorder (BIID; First, 2005; First & Fisher, 2012) or even conceptualized as a paraphilia and denoted apotemnophilia (Brang et al., 2008; Ramachandran & McGeoch, 2007), we suggest that xenomelia can be conceptualized as a neurological disorder that is free of any of the negative connotations associated with BIID and apotemnophilia as has been already suggested by others elsewhere (McGeoch et al., 2011). However, in contrast to McGeoch et al. (2011), who suggested that xenomelia might be a right parietal lobe syndrome, we were able to show that in addition to the right parietal lobe and right SI, SII, and the insula (Hilti et al., 2013), regions which have been shown to be hyperconnected in this study, several further key players of the sensorimotor system such as the PCL (housing SI leg representations), SMA, basal ganglia, cerebellum, and premotor cortex also revealed structural and functional hyperconnectivity in xenomelia compared with healthy control men.

### 4.2 Structural hyperconnectivity in xenomelia

Our structural whole‐brain connectome analysis using network‐based statistics (Zalesky et al., 2010) revealed a subnetwork with enhanced connectivity in xenomelia compared with control men (Figure 1). When focusing on the hyperconnected nodes, it is quite impressive that most of the affected nodes belong to the sensorimotor system (see Table 2). Of the 35 connections distributed over 33 nodes shown in solution 1 of the 116‐node network analysis (Figure 1a), 17 connections have both nodes within the sensorimotor system, 16 connections have at least one node within the sensorimotor system, whereas only two connections were between regions not predicted a priori. Furthermore, most of the cortical nodes affected are located within the right hemisphere, whereas the subcortical nodes pallidum, caudate, putamen, and thalamus were more often affected in the left than the right hemisphere. This finding is in line with our subcortical shape analyses that revealed a preponderance of tissue displacements in left compared with right subcortical structures (Hänggi et al., 2016). The cerebellum, PCL, and SMA are the nodes with the highest degree of affected connections when inspecting solution 2 of the 116‐node network analysis (Figure 1b), and all these nodes except one are within the right hemisphere (connections printed in bold in Table 2).

In contrast to the two subnetworks found with the 116‐node network analyses that showed clear lateralization to the right hemisphere (see above), the subnetwork found in the 28‐node xenomelia‐specific network analysis is less lateralized to the right and, more important, showed only a statistical trend toward significance. However, when looking at the three most important cortical brain regions that have been found to be altered in xenomelia with respect to cortical thickness and surface area (Hilti et al., 2013), that is, the SI (four connections) and SII (two connections) and the SPL (two connections), structural connectivity of the 28‐node xenomelia‐specific network analysis was enhanced only in the right‐hemispheric representations of these three xenomelia‐related core brain regions.

Taken together, our structural connectome analyses provide strong evidence that in addition to the structural and functional anomalies of specific brain regions in xenomelia already reported in the literature (Blom et al., 2016; Hänggi et al., 2016; Hilti et al., 2013; McGeoch et al., 2011; van Dijk et al., 2013), the number of reconstructed streamlines from/to these brain regions is strongly increased in men with compared to without xenomelia, suggesting that these regions are hyperconnected to other brain regions inside and outside of the sensorimotor system. The network solution 1 and 2 of the 116‐node network analyses revealed that a lot of the connections showing structural hyperconnectivity have nodes located within the cerebellum, including both the cerebellar hemispheres as well as the vermis.

With respect to the specificity of the findings, the nodes found to be hyperconnected in xenomelia in network solution 2 (Figure 1b and connections printed in bold in Table 2) showed high specificity because 21 of 22 nodes were within the sensorimotor system. One node, the right frontal operculum is considered to be a premotor region in the broader sense as well and could therefore also be accounted for the sensorimotor system. The frontal operculum is importantly involved in the rubber hand illusion (Ehrsson, Spence, & Passingham, 2004), which consists in a distorted sense of self of one particular body part (Lenggenhager, Hilti, & Brugger, 2015). More broadly speaking, the same region is involved in the construction and maintenance of a coherent representation of the entire body (Moseley et al., 2012; Tsakiris, Hesse, Boy, Haggard, & Fink, 2007), a representation arguably disturbed in xenomelia. The right PCL showed three pathways to/from the cerebellum that were hyperconnected, and a cluster with an anomaly small cortical surface area in xenomelia has already been reported in this brain region for the same participants (Hilti et al., 2013). It is important to realize that the primary somatosensory and primary motor representation of the left leg, that is, the body part most frequently desired to be amputated in xenomelia in general and specifically in our sample, are exactly represented in this particular brain region of the right PCL.

One of the most interesting nodes related to xenomelia within the sensorimotor network is a certain area in the right SPL (Hilti et al., 2013; McGeoch et al., 2011; van Dijk et al., 2013) and this region has been suggested to be the highest region of integration with respect to the concepts of body scheme, image, or even body matrix (Moseley et al., 2012). In the 116‐network analyses, neither the right nor the left SPL showed to be hyperconnected to any other nodes in the whole‐brain network in xenomelia. This might be related to the fact that the SPL is a relatively large brain region and the area of differences reported within the SPL is rather small (Hilti et al., 2013; McGeoch et al., 2011). In the xenomelia‐specific network analysis (28 nodes), however, where the node SPL has been constructed using a sphere around the coordinates reported in the literature (Hilti et al., 2013), two pathways from/to the right SPL were hyperconnected, the stronger connection is running to/from the right caudate nucleus and the weaker connection is running to/from the left caudate nucleus (Table 2).

### 4.3 Functional hyperconnectivity in xenomelia

Our functional network analyses followed the same approach as reported above for the structural ones; hence, we first report the findings from the 116‐node network analyses followed by the xenomelia‐specific 28‐node network analysis. In addition, we constructed a functional subnetwork restricted to the connections that have been shown to be structurally hyperconnected (see Figure 1a, Table 2). When focusing on the hyperconnected nodes, a predominant involvement of the sensorimotor system is less impressive (see Table 4) compared to the structural network findings. Of the 24 connections distributed over 20 nodes shown in solution 1 of the 116‐node network analysis (Figure 3a), 14 connections were found among nodes belonging to the sensorimotor system with a preponderance of cerebellar nodes, but also including the right SMA, right PCL, right PoCG as well as the left and right precentral gyrus.

In contrast to the findings from the structural network analyses (see Figures 1 and 2, Tables 2 and 3), the connections with increased functional connectivity derived from the 116‐node functional network analyses are less lateralized to the right hemisphere (see Figure 3, Table 4). The nodes with the highest degrees in the 116‐node functional network analyses are located in the middle part of the cingulate cortex bilaterally (10 connections) and in the cerebellum (9 connections). Although we did not formulate any a priori hypothesis with respect to the cingulate cortex, we note that the cingulate motor area, located ventrally adjacent to the SMA, is housed within the middle part of the cingulate cortex (Brazdil, Kuba, & Rektor, 2006; Liberg et al., 2014; Ullsperger & von Cramon, 2003). This cingulate motor area is also part of the sensorimotor system, and it should therefore not come as a surprise that it evidenced an increased functional connectivity in our xenomelia group.

### 4.4 Associations between structural and functional connectivity strength of the altered networks

The correlations between structural and functional connectivity strengths of the hyperconnected networks were all positive suggesting that the two different types of connectivity alterations are related, that is, the stronger the structural connections the stronger the functional ones. Although this evidence is based on correlations only that do not provide any information with respect to causality, it is conceivable that functional hyperconnectivity results rather from structural hyperconnectivity than the other way around. However, if structural hyperconnectivity has the same potential to break down a network's functioning as has a structurally hypoconnected network, then functional hyperconnectivity might be interpreted as a sign of functional compensation in response to the structural network impairments. Exactly this pattern of connectivity dissociation has been reported previously in healthy subjects notably in fronto‐parietal networks (Eickhoff et al., 2010) and in pathological conditions such as major and late life depression (de Kwaasteniet et al., 2013; Steffens, Taylor, Denny, Bergman, & Wang, 2011; Wu et al., 2011) and stroke (Zhang et al., 2014). Alternatively, however, an increase in functional connectivity may reflect a pathological loss of inhibitory neural activity within structurally damaged cortical networks as has been shown in patients suffering from amyotrophic lateral sclerosis (Douaud, Filippini, Knight, Talbot, & Turner, 2011). For clarifying the causal relationship of the connectivity differences, further research must be performed using longitudinal study designs.

### 4.5 Neurobiological mechanisms of hyperconnectivity

The desire for healthy limb amputation might be caused by a disturbed representation of the body at different levels of sensorimotor integration. Among the factors with a perturbation potential are cortical regions showing reduced cortical thickness or surface area of (Hilti et al., 2013) or subcortical regions showing altered striatal, pallidal, and thalamic shape (Hänggi et al., 2016), but also altered connectivities in networks responsible for the construction and maintenance of a coherent body image (findings of this study). Altered structural and functional network connectivities have also been reported for a variety of psychiatric diseases (Cao, Wang, & He, 2015) and it has been suggested that, at least for functional brain networks, hyperconnectivity is a fundamental response to (focal) neurological disruption (Hillary et al., 2014, 2015).

However, the question of when and how the structural and functional hyperconnectivity uncovered in the present investigation has been established in the first line is not easy to answer. We may only speculate about potential neurobiological mechanisms underlying the hyperconnectivity patterns observed in xenomelia in this study.

One such potential mechanism may be a failure in neural pruning, that is, an insufficient elimination of unneeded neural populations and connections. Such pruning processes are driven either by apoptosis (Cusack, Swahari, Hampton Henley, Michael Ramsey, & Deshmukh, 2013) or by experience (Eyding, Schweigart, & Eysel, 2002; Yu et al., 2013), but in all likelihood by a combination of both factors. It is well known from neurophysiological studies that the rapid phase of synaptogenesis in early childhood is followed by subsequent longer periods of pruning, during which synapses are eliminated by about 40% to reach near‐mature levels at 11 years of age (Huttenlocher, 1984, 1990; Huttenlocher & de Courten, 1987; Huttenlocher, de Courten, Garey, & Van der Loos, 1982). For example, it has been suggested that sensory deprivation during early childhood might cause a substantial reduction in pruning of the exuberant cortico‐cortical and/or cortico‐thalamo‐cortical connections that exist in early infancy. This reduced pruning due to sensory deprivation will result in a greater survival of the exuberant connections and, consequently, a thicker visual cortex. This has been suggested for early or congenitally blind subjects in whom visual cortex was found to be thicker than in sighted control subjects (Anurova, Renier, De Volder, Carlson, & Rauschecker, 2015). It is conceivable that reduced pruning, whatever its ultimate causes may be, results in a white matter hyperconnectivity in participants suffering from xenomelia.

In what the deprivation during early development could consist in the case of xenomelia, is unclear. We would expect a lack of regular somatosensory input from and/or an underuse of the limb later considered not belonging, but persons with xenomelia do not systematically report such things. Some unspecific impoverishment during early childhood has occasionally been mentioned (Riordan & Appleby, 1994), but seems an unlikely candidate to account for the rejection of one particular limb as a consequence of aberrant neurogenesis.

The nodes of the sensorimotor system are structurally hyperconnected to each other and to nodes outside of the sensorimotor system as shown in this study, for which structural connectivity was operationalized as the number of reconstructed streamlines between two nodes. This number is related to the number and/or volume of the real axonal pathways. Because streamlines are more abundant among sensorimotor nodes in xenomelia compared with the control group, this increase in structural connectivity can explain the functional hyperconnectivity among those sensorimotor nodes. If there are more axons, or axons have a larger diameter between sensorimotor nodes in xenomelia, either more information is propagated between those nodes or information transfer is faster, which in turn has the potential to result in increased functional connectivity. Such enhanced functional connectivity is capable of producing a breakdown in the normal functioning (Hillary et al., 2014, 2015) of the sensorimotor system, may by inference also hamper establishing a proper body image, and may ultimately lead to the feeling that a body part is not belonging to the rest of the body.

### 4.6 Limitations

Several limitations of this study are worth mentioning. First, the sample size (13 participants with xenomelia plus an equal number of control participants) is rather small. However, we have gone a long way to find enough persons presenting with the condition, yet belonging to an adequately homogenous group of nonpsychotic individuals, who do not display any self‐injurious behavior, do not suffer from body‐dysmorphic disorder, and whose amputation desire is not primarily sexually motivated. Second, because of the small sample size, it was not possible to build subgroups to investigate, for instance, differences with respect to whether the amputation desire targeted the left leg, the right leg, or both legs. Whether the pattern of hyperconnectivity observed in this study changes if more individuals with a right‐sided amputation desire are included remains to be shown in future studies that should recruit larger samples to address the issue of lateralization explicitly. It might be difficult, however, to recruit enough subjects with a right leg amputation desire due to the preponderance of the left leg as the amputation target. Third, we investigated men only; again, given the rarity of the condition and the fact that an overwhelming majority of persons affected are male (only about 10% of people affected are female; Blom, Hennekam, & Denys, 2012) this gender bias may be excused. Fourth, although we tested the networks across a range of set thresholds and corrected each single threshold for multiple comparisons, we did not additionally adjust the alpha error probability for the number of set thresholds tested. However, a Bonferroni correction would be too conservative in this context. Fifth, the analysis of the structural 28‐node xenomelia‐specific network revealed an effect only significant on a trend level (*p* = .087, corrected) and some of the brain regions of the 28‐node network were selected nonindependently, that is, based on the same experimental subjects as investigated in previous studies (Hänggi et al., 2016; Hilti et al., 2013). This finding must be interpreted cautiously, therefore, and our study should be taken as a springboard to more extended examinations. Finally, this study is based on cross‐sectional data and therefore it remains unclear whether the structural and functional hyperconnectivity evident within the sensorimotor system are the causes of xenomelia or rather the consequence of the lifelong persistent desire for limb amputation and its associated experiences.

## 5 Conclusions

Proponents of a neurological nature of xenomelia have suggested the right SPL as the key node affected within a brain network responsible for body ownership (Brang et al., 2008; Hilti et al., 2013; McGeoch et al., 2011; van Dijk et al., 2013). This network of body ownership or body image (Moseley et al., 2012) encompasses brain regions of sensorimotor relevance, such as the SI, SII, basal ganglia, thalamus, and premotor cortex (Blom et al., 2016; Hänggi et al., 2016; Hilti et al., 2013; van Dijk et al., 2013). This study extends these previous findings by showing that, in addition to the focal structural alterations in the above mentioned brain structures, structural and functional connectivity between the single parts of this network are markedly increased in xenomelia. Our study provides strong empirical evidence of structural and functional hyperconnectivity in the sensorimotor system in individuals with xenomelia, comprising those regions that are core for the experience of a unified bodily self. Structural and functional brain hyperconnectivity is a common response to focal neurological disruption and affects brain regions differentially (Hillary et al., 2014, 2015) as observed here within the somatosensory system in xenomelia. Brain hyperconnectivity might account for the limb dis‐ownership commonly reported by xenomelia subjects. We speculate that one phenomenal correlate of this hyperconnectivity might be a pathological “over‐attention” to specific body parts and the feeling of “over‐completeness”, culminating, in some individuals, in a desire for healthy limb amputation. We emphasize, however, that the present findings must be interpreted cautiously due to the small sample size.

## Conflict of Interest

All authors declare that there are no competing interests.

## References

[brb3657-bib-0001] Anurova, I. , Renier, L. A. , De Volder, A. G. , Carlson, S. , & Rauschecker, J. P. (2015). Relationship between cortical thickness and functional activation in the early blind. Cerebral Cortex, 25, 2035–2048. 24518755 10.1093/cercor/bhu009 PMC4494021

[brb3657-bib-0002] Aoyama, A. , Krummenacher, P. , Palla, A. , Hilti, L. M. , & Brugger, P. (2011). Impaired spatial‐temporal integration of touch in xenomelia (body integrity identity disorder). Spatial Cognition & Computation, 12, 96–110.

[brb3657-bib-0003] Behrens, T. E. J. , Woolrich, M. W. , Jenkinson, M. , Johansen‐Berg, H. , Nunes, R. G. , Clare, S. , … Smith, S. M. (2003). Characterization and propagation of uncertainty in diffusion‐weighted MR imaging. Magnetic Resonance in Medicine, 50, 1077–1088. 14587019 10.1002/mrm.10609

[brb3657-bib-0004] Berlucchi, G. , & Aglioti, S. M. (2010). The body in the brain revisited. Experimental Brain Research, 200, 25–35. 19690846 10.1007/s00221-009-1970-7

[brb3657-bib-0005] Blanke, O. , Morgenthaler, F. D. , Brugger, P. , & Overney, L. S. (2009). Preliminary evidence for a fronto‐parietal dysfunction in able‐bodied participants with a desire for limb amputation. Journal of Neuropsychology, 3, 181–200. 19338723 10.1348/174866408X318653

[brb3657-bib-0006] Blom, R. M. , Hennekam, R. C. , & Denys, D. (2012). Body integrity identity disorder. PLoS ONE, 7, e34702. 22514657 10.1371/journal.pone.0034702 PMC3326051

[brb3657-bib-0007] Blom, R. M. , van Wingen, G. A. , van der Wal, S. J. , Luigjes, J. , van Dijk, M. T. , Scholte, H. S. , & Denys, D. (2016). The desire for amputation or paralyzation: Evidence for structural brain anomalies in body integrity identity disorder (BIID). PLoS ONE, 11, e0165789. 27832097 10.1371/journal.pone.0165789 PMC5104450

[brb3657-bib-0008] Brang, D. , McGeoch, P. D. , & Ramachandran, V. S. (2008). Apotemnophilia: A neurological disorder. NeuroReport, 19, 1305–1306. 18695512 10.1097/WNR.0b013e32830abc4d

[brb3657-bib-0009] Brazdil, M. , Kuba, R. , & Rektor, I. (2006). Rostral cingulate motor area and paroxysmal alien hand syndrome. Journal of Neurology, Neurosurgery and Psychiatry, 77, 992–993. 10.1136/jnnp.2005.082529 PMC2077630 16844957

[brb3657-bib-0010] Brugger, P. (2007). Hostile interactions between body and self. Dialogues in Clinical Neuroscience, 9, 210–213.

[brb3657-bib-0011] Brugger, P. , & Lenggenhager, B. (2014). The bodily self and its disorders: Neurological, psychological and social aspects. Current Opinion in Neurology, 27, 644–652. 25333602 10.1097/WCO.0000000000000151

[brb3657-bib-0012] Brugger, P. , Lenggenhager, B. , & Giummarra, M. J. (2013). Xenomelia: A social neuroscience view of altered bodily self‐consciousness. Frontiers in Psychology, 4, 204. 23630513 10.3389/fpsyg.2013.00204 PMC3634160

[brb3657-bib-0013] Bullmore, E. T. , Suckling, J. , Overmeyer, S. , Rabe‐Hesketh, S. , Taylor, E. , & Brammer, M. J. (1999). Global, voxel, and cluster tests, by theory and permutation, for a difference between two groups of structural MR images of the brain. IEEE Transactions on Medical Imaging, 18, 32–42. 10193695 10.1109/42.750253

[brb3657-bib-0014] Cao, M. , Wang, Z. , & He, Y. (2015). Connectomics in psychiatric research: Advances and applications. Neuropsychiatric Disease and Treatment, 11, 2801–2810. 26604764 10.2147/NDT.S63470 PMC4631424

[brb3657-bib-0015] Chao‐Gan, Y. , & Yu‐Feng, Z. (2010). DPARSF: A MATLAB toolbox for “pipeline” data analysis of resting‐state fMRI. Frontiers in Systems Neuroscience, 4, 13. 20577591 10.3389/fnsys.2010.00013 PMC2889691

[brb3657-bib-0016] Critchely, M. (1953). The parietal lobes. London: Eward Arnold.

[brb3657-bib-0017] Cusack, C. L. , Swahari, V. , Hampton Henley, W. , Michael Ramsey, J. , & Deshmukh, M. (2013). Distinct pathways mediate axon degeneration during apoptosis and axon‐specific pruning. Nature Communications, 4, 1876. 10.1038/ncomms2910 PMC4183061 23695670

[brb3657-bib-0018] de Kwaasteniet, B. , Ruhe, E. , Caan, M. , Rive, M. , Olabarriaga, S. , Groefsema, M. , … Denys, D. (2013). Relation between structural and functional connectivity in major depressive disorder. Biological Psychiatry, 74, 40–47. 23399372 10.1016/j.biopsych.2012.12.024

[brb3657-bib-0019] Douaud, G. , Filippini, N. , Knight, S. , Talbot, K. , & Turner, M. R. (2011). Integration of structural and functional magnetic resonance imaging in amyotrophic lateral sclerosis. Brain, 134, 3470–3479. 22075069 10.1093/brain/awr279

[brb3657-bib-0020] Ehrsson, H. H. , Spence, C. , & Passingham, R. E. (2004). That's my hand! Activity in premotor cortex reflects feeling of ownership of a limb. Science, 305, 875–877. 15232072 10.1126/science.1097011

[brb3657-bib-0021] Eickhoff, S. B. , Jbabdi, S. , Caspers, S. , Laird, A. R. , Fox, P. T. , Zilles, K. , & Behrens, T. E. (2010). Anatomical and functional connectivity of cytoarchitectonic areas within the human parietal operculum. The Journal of Neuroscience, 30, 6409–6421. 20445067 10.1523/JNEUROSCI.5664-09.2010 PMC4791040

[brb3657-bib-0022] Eyding, D. , Schweigart, G. , & Eysel, U. T. (2002). Spatio‐temporal plasticity of cortical receptive fields in response to repetitive visual stimulation in the adult cat. Neuroscience, 112, 195–215. 12044484 10.1016/s0306-4522(02)00039-8

[brb3657-bib-0023] First, M. B. (2005). Desire for amputation of a limb: Paraphilia, psychosis, or a new type of identity disorder. Psychological Medicine, 35, 919–928. 15997612 10.1017/s0033291704003320

[brb3657-bib-0024] First, M. B. , & Fisher, C. E. (2012). Body integrity identity disorder: The persistent desire to acquire a physical disability. Psychopathology, 45, 3–14. 22123511 10.1159/000330503

[brb3657-bib-0025] Giummarra, M. J. , Gibson, S. J. , Georgiou‐Karistianis, N. , & Bradshaw, J. L. (2008). Mechanisms underlying embodiment, disembodiment and loss of embodiment. Neuroscience and Biobehavioral Reviews, 32, 143–160. 17707508 10.1016/j.neubiorev.2007.07.001

[brb3657-bib-0026] Hänggi, J. , Bellwald, D. , & Brugger, P. (2016). Shape alterations of basal ganglia and thalamus in xenomelia. NeuroImage: Clinical, 11, 760–769. 27330976 10.1016/j.nicl.2016.05.015 PMC4909827

[brb3657-bib-0027] Hayasaka, S. , & Nichols, T. E. (2004). Combining voxel intensity and cluster extent with permutation test framework. NeuroImage, 23, 54–63. 15325352 10.1016/j.neuroimage.2004.04.035

[brb3657-bib-0028] Hillary, F. G. , Rajtmajer, S. M. , Roman, C. A. , Medaglia, J. D. , Slocomb‐Dluzen, J. E. , Calhoun, V. D. , … Wylie, G. R. (2014). The rich get richer: Brain injury elicits hyperconnectivity in core subnetworks. PLoS ONE, 9, e104021. 25121760 10.1371/journal.pone.0104021 PMC4133194

[brb3657-bib-0029] Hillary, F. G. , Roman, C. A. , Venkatesan, U. , Rajtmajer, S. M. , Bajo, R. , & Castellanos, N. D. (2015). Hyperconnectivity is a fundamental response to neurological disruption. Neuropsychology, 29, 59–75. 24933491 10.1037/neu0000110

[brb3657-bib-0030] Hilti, L. M. , Hänggi, J. , Vitacco, D. A. , Kraemer, B. , Palla, A. , Luechinger, R. , … Brugger, P. (2013). The desire for healthy limb amputation: Structural brain correlates and clinical features of xenomelia. Brain, 136, 318–329. 23263196 10.1093/brain/aws316

[brb3657-bib-0031] Huttenlocher, P. R. (1984). Synapse elimination and plasticity in developing human cerebral cortex. American Journal of Mental Deficiency, 88, 488–496. 6731486

[brb3657-bib-0032] Huttenlocher, P. R. (1990). Morphometric study of human cerebral cortex development. Neuropsychologia, 28, 517–527. 2203993 10.1016/0028-3932(90)90031-i

[brb3657-bib-0033] Huttenlocher, P. R. , & de Courten, C. (1987). The development of synapses in striate cortex of man. Human Neurobiology, 6, 1–9. 3583840

[brb3657-bib-0034] Huttenlocher, P. R. , de Courten, C. , Garey, L. J. , & Van der Loos, H. (1982). Synaptogenesis in human visual cortex–evidence for synapse elimination during normal development. Neuroscience Letters, 33, 247–252. 7162689 10.1016/0304-3940(82)90379-2

[brb3657-bib-0035] Johnson, A. J. , Liew, S.‐L. , & Aziz‐Zadeh, L. (2011). Demographics, learning and imitation, and body schema in body integrity identity disorder. Indiana University Undergraduate Journal of Cognitive Science, 6, 8–18.

[brb3657-bib-0036] Lenggenhager, B. , Hilti, L. , & Brugger, P. (2015). Disturbed body integrity and the “rubber foot illusion”. Neuropsychology, 29, 205–211. 25265068 10.1037/neu0000143

[brb3657-bib-0037] Liberg, B. , Klauser, P. , Harding, I. H. , Adler, M. , Rahm, C. , Lundberg, J. , … Wahlund, B. (2014). Functional and structural alterations in the cingulate motor area relate to decreased fronto‐striatal coupling in major depressive disorder with psychomotor disturbances. Frontiers in Psychiatry, 5, 176. 25538633 10.3389/fpsyt.2014.00176 PMC4255491

[brb3657-bib-0038] Loetscher, T. , Regard, M. , & Brugger, P. (2006). Misoplegia: A review of the literature and a case without hemiplegia. Journal of Neurology, Neurosurgery & Psychiatry, 77, 1099–1100. 10.1136/jnnp.2005.087163 PMC2077726 16914766

[brb3657-bib-0039] McGeoch, P. D. , Brang, D. , Song, T. , Lee, R. R. , Huang, M. , & Ramachandran, V. S. (2011). Xenomelia: A new right parietal lobe syndrome. Journal of Neurology, Neurosurgery and Psychiatry, 82, 1314–1319. 10.1136/jnnp-2011-300224 21693632

[brb3657-bib-0040] Moseley, G. L. , Gallace, A. , & Spence, C. (2012). Bodily illusions in health and disease: Physiological and clinical perspectives and the concept of a cortical ‘body matrix’. Neuroscience and Biobehavioral Reviews, 36, 34–46. 21477616 10.1016/j.neubiorev.2011.03.013

[brb3657-bib-0041] Nichols, T. E. , & Holmes, A. P. (2002). Nonparametric permutation tests for functional neuroimaging: A primer with examples. Human Brain Mapping, 15, 1–25. 11747097 10.1002/hbm.1058 PMC6871862

[brb3657-bib-0042] Park, J. H. , Park, S. W. , Kang, S. H. , Nam, T. K. , Min, B. K. , & Hwang, S. N. (2009). Detection of traumatic cerebral microbleeds by susceptibility‐weighted image of MRI. Journal of Korean Neurosurgical Society, 46, 365–369. 19893728 10.3340/jkns.2009.46.4.365 PMC2773396

[brb3657-bib-0043] Qing, Z. , Dong, Z. , Li, S. , Zang, Y. , & Liu, D. (2015). Global signal regression has complex effects on regional homogeneity of resting state fMRI signal. Magnetic Resonance Imaging, 33, 1306–1313. 26234499 10.1016/j.mri.2015.07.011

[brb3657-bib-0044] Ramachandran, V. S. , & McGeoch, P. (2007). Can vestibular caloric stimulation be used to treat apotemnophilia? Medical Hypotheses, 69, 250–252. 17292561 10.1016/j.mehy.2006.12.013

[brb3657-bib-0045] Riordan, D. , & Appleby, L. (1994). A man who does not use his arms. The British Journal of Psychiatry, 165, 123. 7953022 10.1192/bjp.165.1.123

[brb3657-bib-0046] Smith, S. M. , Jenkinson, M. , Woolrich, M. W. , Beckmann, C. F. , Behrens, T. E. , Johansen‐Berg, H. , … Matthews, P. M. (2004). Advances in functional and structural MR image analysis and implementation as FSL. NeuroImage, 23(Suppl 1), S208–S219. 15501092 10.1016/j.neuroimage.2004.07.051

[brb3657-bib-0047] Steffens, D. C. , Taylor, W. D. , Denny, K. L. , Bergman, S. R. , & Wang, L. (2011). Structural integrity of the uncinate fasciculus and resting state functional connectivity of the ventral prefrontal cortex in late life depression. PLoS ONE, 6, e22697. 21799934 10.1371/journal.pone.0022697 PMC3142185

[brb3657-bib-0048] Tsakiris, M. , Hesse, M. D. , Boy, C. , Haggard, P. , & Fink, G. R. (2007). Neural signatures of body ownership: A sensory network for bodily self‐consciousness. Cerebral Cortex, 17, 2235–2244. 17138596 10.1093/cercor/bhl131

[brb3657-bib-0049] Tzourio‐Mazoyer, N. , Landeau, B. , Papathanassiou, D. , Crivello, F. , Etard, O. , Delcroix, N. , … Joliot, M. (2002). Automated anatomical labeling of activations in SPM using a macroscopic anatomical parcellation of the MNI MRI single‐subject brain. NeuroImage, 15, 273–289. 11771995 10.1006/nimg.2001.0978

[brb3657-bib-0050] Ullsperger, M. , & von Cramon, D. Y. (2003). Error monitoring using external feedback: Specific roles of the habenular complex, the reward system, and the cingulate motor area revealed by functional magnetic resonance imaging. The Journal of Neuroscience, 23, 4308–4314. 12764119 10.1523/JNEUROSCI.23-10-04308.2003 PMC6741115

[brb3657-bib-0051] Vallar, G. , & Ronchi, R. (2009). Somatoparaphrenia: A body delusion. A review of the neuropsychological literature. Experimental Brain Research, 192, 533–551. 18813916 10.1007/s00221-008-1562-y

[brb3657-bib-0052] van Dijk, M. T. , van Wingen, G. A. , van Lammeren, A. , Blom, R. M. , de Kwaasteniet, B. P. , Scholte, H. S. , & Denys, D. (2013). Neural basis of limb ownership in individuals with body integrity identity disorder. PLoS ONE, 8, e72212. 23991064 10.1371/journal.pone.0072212 PMC3749113

[brb3657-bib-0053] Wong, C. W. , Olafsson, V. , Tal, O. , & Liu, T. T. (2012). Anti‐correlated networks, global signal regression, and the effects of caffeine in resting‐state functional MRI. NeuroImage, 63, 356–364. 22743194 10.1016/j.neuroimage.2012.06.035 PMC3444518

[brb3657-bib-0054] Wu, M. , Andreescu, C. , Butters, M. A. , Tamburo, R. , Reynolds, C. F. 3rd , & Aizenstein, H. (2011). Default‐mode network connectivity and white matter burden in late‐life depression. Psychiatry Research, 194, 39–46. 21824753 10.1016/j.pscychresns.2011.04.003 PMC3189685

[brb3657-bib-0055] Xia, M. , Wang, J. , & He, Y. (2013). BrainNet Viewer: A network visualization tool for human brain connectomics. PLoS ONE, 8, e68910. 23861951 10.1371/journal.pone.0068910 PMC3701683

[brb3657-bib-0056] Yeh, C. J. , Tseng, Y. S. , Lin, Y. R. , Tsai, S. Y. , & Huang, T. Y. (2015). Resting‐state functional magnetic resonance imaging: The impact of regression analysis. Journal of Neuroimaging, 25, 117–123. 24571121 10.1111/jon.12085

[brb3657-bib-0057] Yu, X. , Wang, G. , Gilmore, A. , Yee, A. X. , Li, X. , Xu, T. , … Zuo, Y. (2013). Accelerated experience‐dependent pruning of cortical synapses in ephrin‐A2 knockout mice. Neuron, 80, 64–71. 24094103 10.1016/j.neuron.2013.07.014 PMC3792401

[brb3657-bib-0058] Zalesky, A. , Fornito, A. , & Bullmore, E. T. (2010). Network‐based statistic: Identifying differences in brain networks. NeuroImage, 53, 1197–1207. 20600983 10.1016/j.neuroimage.2010.06.041

[brb3657-bib-0059] Zhang, J. , Meng, L. , Qin, W. , Liu, N. , Shi, F. D. , & Yu, C. (2014). Structural damage and functional reorganization in ipsilesional m1 in well‐recovered patients with subcortical stroke. Stroke, 45, 788–793. 24496396 10.1161/STROKEAHA.113.003425

