# Supplementary material for: Structural and functional hyperconnectivity within the sensorimotor system in xenomelia
Source: Brain Behav. 2017 Feb 23;7(3):e00657. doi: 10.1002/brb3.657 (PMC5346531; doi:10.1002/brb3.657)
Supplement: Supplementary file 1 [file BRB3-7-e00657-s001.docx]

**Structural and functional hyperconnectivity within the sensorimotor system in xenomelia**

Jürgen Hänggi^a^, Deborah A. Vitacco^b^, Leonie M. Hilti^b^, Roger Luechinger^c^, Bernd Kraemer^d^, Peter Brugger^b,e^

^a^ Division Neuropsychology, Department of Psychology, University of Zurich, Zurich, Switzerland

^b^ Neuropsychology Unit, Department of Neurology, University Hospital Zurich, Zurich, Switzerland

^c^ Institute for Biomedical Engineering, University and ETH Zurich, Zurich, Switzerland

^d^ Psychiatric Services, Hospitals of the Canton of Solothurn, Olten, Switzerland

^e^ Center for Integrative Human Physiology (ZIHP), University of Zurich, Zurich, Switzerland

Corresponding authors:

Jürgen Hänggi, Division Neuropsychology, Department of Psychology, University of Zurich, Binzmühlestrasse 14 / P.O. 25 CH-8090 Zurich, Switzerland

Phone: +41 44 635 7397, Fax: +41 44 635 7409, E-mail: j.haenggi@psychologie.uzh.ch

Peter Brugger, Neuropsychological Unit, Department of Neurology, University Hospital Zurich, Rämistrasse 100, CH-8091 Zurich, Switzerland

Phone: +41 44 255 5570, Fax: +41 44 255 1260, E-mail: peter.brugger@usz.ch

Address reprint requests to Prof. Dr. Brugger at peter.brugger@usz.ch

**
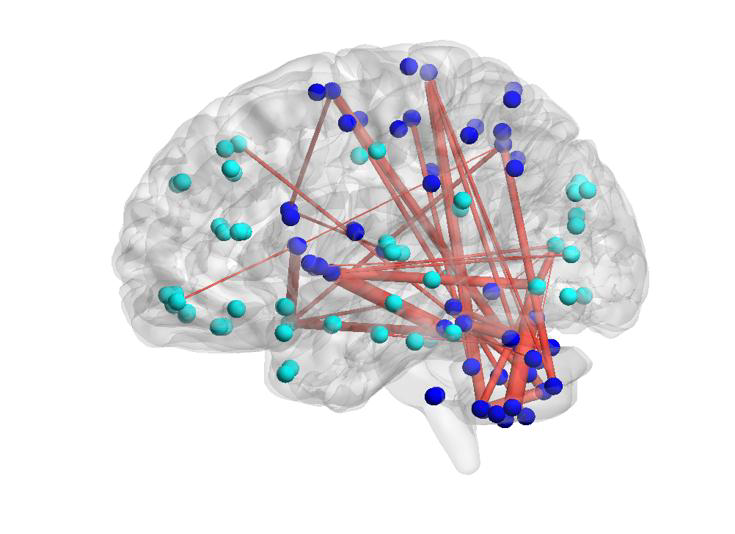
Supplementary animations**

**Supplementary animation 1.** Increased structural connectivity in the sensorimotor system in patients suffering from xenomelia. For details please see Fig. 1A (solution 1) and Table 2 in the main manuscript.

**
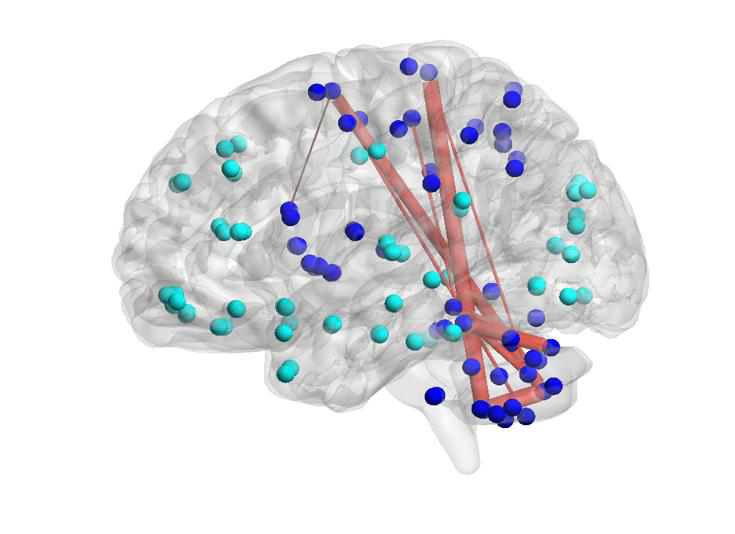
**

**Supplementary animation 2.** Increased structural connectivity in the sensorimotor system in patients suffering from xenomelia. For details please see Fig. 1B (solution 2) and Table 2 in the main manuscript.

**
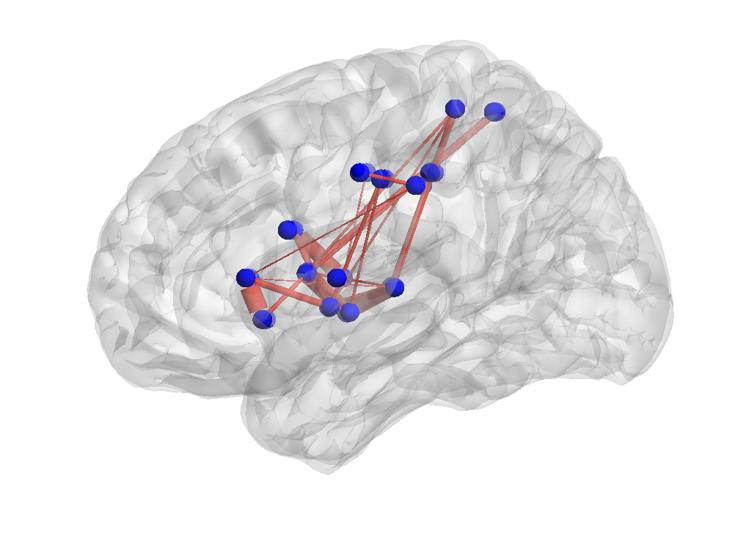
**

**Supplementary animation 3.** Increased structural connectivity in the sensorimotor system in patients suffering from xenomelia. For details please see Fig. 2 and Table 3 in the main manuscript.

**
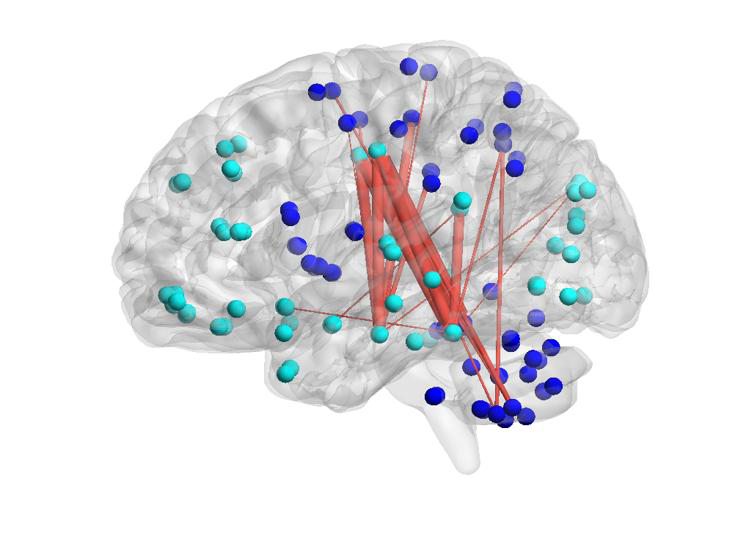
**

**Supplementary animation 4.** Increased functional connectivity in the sensorimotor system in patients suffering from xenomelia. For details please see Fig. 3A (solution 1) and Table 4 in the main manuscript.

**
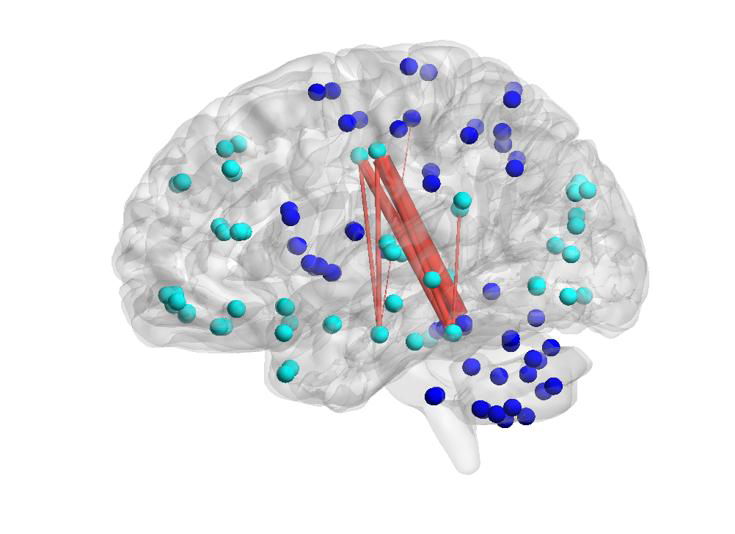
**

**Supplementary animation 5.** Increased functional connectivity in the sensorimotor system in patients suffering from xenomelia. For details please see Fig. 3B (solution 2) and Table 4 in the main manuscript.

**
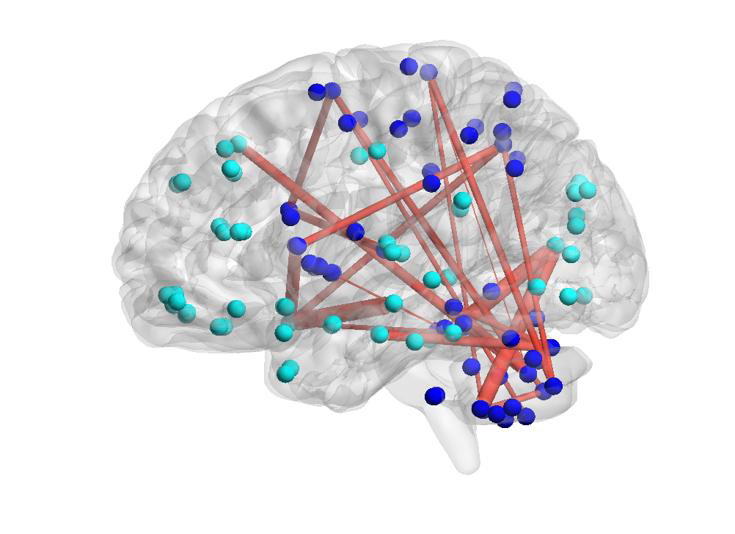
**

**Supplementary animation 6.** Increased functional connectivity in the sensorimotor system in patients suffering from xenomelia. Network analysis has been restricted to the connections showing structural hyperconnectivity. For details please see Fig. 4 and Table 5 in the main manuscript.
